# Relationships between immune cells, blood metabolites, and intrahepatic cholangiocarcinoma: a Mendelian randomization study with experimental validation

**DOI:** 10.3389/fcell.2026.1836249

**Published:** 2026-05-22

**Authors:** Zhipeng Ye, BuLang Tang, Yuanjian Zhang, Hanhan Chen, Jie Li, Zhitao Ye, Xuejian Liu, Di Li

**Affiliations:** 1 Hepatology Unit, Departments of Infectious Disease, Guangzhou Women and Children’s Medical Center, Guangzhou Medical University, Guangzhou, China; 2 The Fifth Affiliated Hospital of Guangzhou Medical University, Guangzhou, China; 3 Guangzhou Women and Children’s Medical Center, Guangzhou Medical University, Guangzhou, China; 4 School of Pharmaceutical Sciences, Guangzhou Medical University, Guangzhou, China; 5 Department of Radiology, Xinjiang 474 Hospital, Urumqi, China; 6 Department of Ultrasonography, Guangzhou Women and Children’s Medical Center, Guangzhou Medical University, Guangzhou, China

**Keywords:** genome-wide association studies, immune cells, intrahepatic cholangiocarcinoma, Mendelian randomization, plasma metabolites

## Abstract

**Introduction:**

Increasing studies have found links between immune traits and intrahepatic cholangiocarcinoma (ICC). However, the presence of confounding variables makes it difficult to determine if this correlation shows a real cause-and-effect relationship.

**Methods:**

To ascertain the genetic association between immune cell profiles and the risk of ICC, we employed two-sample Mendelian randomization (MR) to figure out the potential therapeutic targets. A mediation analysis was performed to identify potential blood metabolite mediators, thereby elucidating the underlying biological mechanisms. The inverse variance weighted method was used as the major analytical tool, with strict sensitivity analyses to account for horizontal pleiotropy and data heterogeneity.

**Results:**

Out of the immunological profiles examined, a trio of unique traits were identified as being highly associated with the risk of ICC. Elevated counts of activated CD4 regulatory T cells (OR = 1.0005, 95% CI = 1.0002–1.0008, p = 0.0013), CD16 on CD14^−^ CD16^+^ monocytes (OR = 1.0003, 95% CI = 1.0001–1.0006, p = 0.0021), and CD16 on CD14^+^ CD16^+^ monocytes (OR = 1.0004, 95% CI = 1.0001–1.0008, p = 0.0087) were identified as risk factors for the development of ICC. Sensitivity analysis revealed no horizontal pleiotropy or heterogeneity. Mediation research reveals that N-acetylleucine and fructosyllysine levels may act as major molecular mediators. Exploratory experiments targeting the DLAT-leucine metabolic axis in HuCC-T1 and RBE cells revealed that disrupting this pathway significantly impairs tumor cell proliferation and migration.

**Conclusion:**

Through two-sample MR analysis, this study demonstrated a causal link between specific immune-cell phenotypes and ICC development. We also identified N-acetylleucine as a vital metabolic link between immune traits and ICC. *In vitro* experiments confirmed these findings. We observed that knocking down DLAT effectively suppressed both the migratory and proliferative potential of ICC cells.

## 1 Introduction

Intrahepatic cholangiocarcinoma (ICC) is the second most common primary liver cancer, which originates from the lining of the bile ducts within the liver. Currently, ICC accounts for 10%–20% of all primary liver malignancies (Zhang et al., 2016). The prevalence of ICC is progressively escalating globally, with the greatest rates recorded in Asian locations, where variables such liver fluke infection contribute to this increase (Banales et al., 2016; Brown et al., 2022). A primary oncogenic environment for the development of ICC is provided by chronic inflammatory processes, which are characterized by biliary stasis and consequential epithelial impairment. The intrahepatic biliary system is substantially predisposed to malignant transformation as a result of persistent inflammation-induced damage to the bile duct lining (Zhang et al., 2016). The sole potentially curative treatment for ICC is surgical resection. However, the five-year survival rate for ICC is still low and varies from 15% to 40% (Bridgewater et al., 2014; Moris et al., 2023; Cui et al., 2025). The poor effectiveness of the present comprehensive treatment approach for ICC highlights the critical need to clarify the pathophysiology and progression mechanisms of ICC.

Emerging data reveals the importance of unique immune cell types in the formation and therapeutic management of various malignancies (Li et al., 2023b; Yu et al., 2023; Mao et al., 2024; Ye et al., 2024). The most recent research path in cancer prevention and treatment is to modify the activity and targeting of different immune cells (Mao et al., 2024). The tumor microenvironment (TME) is a complex ecosystem in which a wide range of specialized immune infiltrates form a highly interacting network. Tumor-associated macrophages (TAMs) and other immune cells perform critical functions in controlling the local microenvironment. Intricate reciprocal interactions between these immune groups and malignant cells create a highly immunosuppressive environment. This specialized niche not only promotes neoplastic development, but also provides resistance, isolating the lesion from host-derived immunosurveillance and eventually cytotoxic clearance (Xu et al., 2024; Ka et al., 2025; Xie et al., 2025). However, the causal link between immune traits and the pathophysiology of ICC is unclear. Circulating plasma metabolites, which operate as major intermediates in endogenous metabolic pathways, are critical signaling mediators that actively manage the clinical course and molecular evolution of many disease disorders. Tan et al. evaluated 614 plasma samples to construct and validate a ten-metabolite signature that consistently stratifies patients with ICC according to risk (Tan et al., 2023). A study showed that patients with IDH1-mutant ICC have higher plasma levels of 2-hydroxyglutarate (Winter et al., 2019). This highlights a link between genetic mutations and metabolite levels. Based on these findings, we hypothesize that immune cells, metabolites, and ICC are causally connected.

Randomized Controlled Trials (RCTs) are considered the gold standard for establishing causal relationships (Li et al., 2023a). However, they are often difficult to conduct due to significant ethical and logistical constraints. Mendelian randomization (MR) offers a robust alternative. It uses genetic variants as instrumental variables to assess the causal impact of exposures on clinical outcomes (Hu et al., 2023; Cho et al., 2023; Zhao et al., 2023). This approach relies on the principle of independent assortment, which mirrors the random allocation used in RCTs. By doing so, MR effectively minimizes the confounding biases (Ference et al., 2021).

In this study, we used a two-sample MR study to investigate the causal relationship between immune traits and ICC. To further explore the potential biological significance of the metabolic pathways identified in our study, we focused on the leucine metabolic axis. Our findings offer a new way to look at the disease and find potential metabolic targets for treatment.

## 2 Methods

### 2.1 Data sources

**FIGURE 1:**
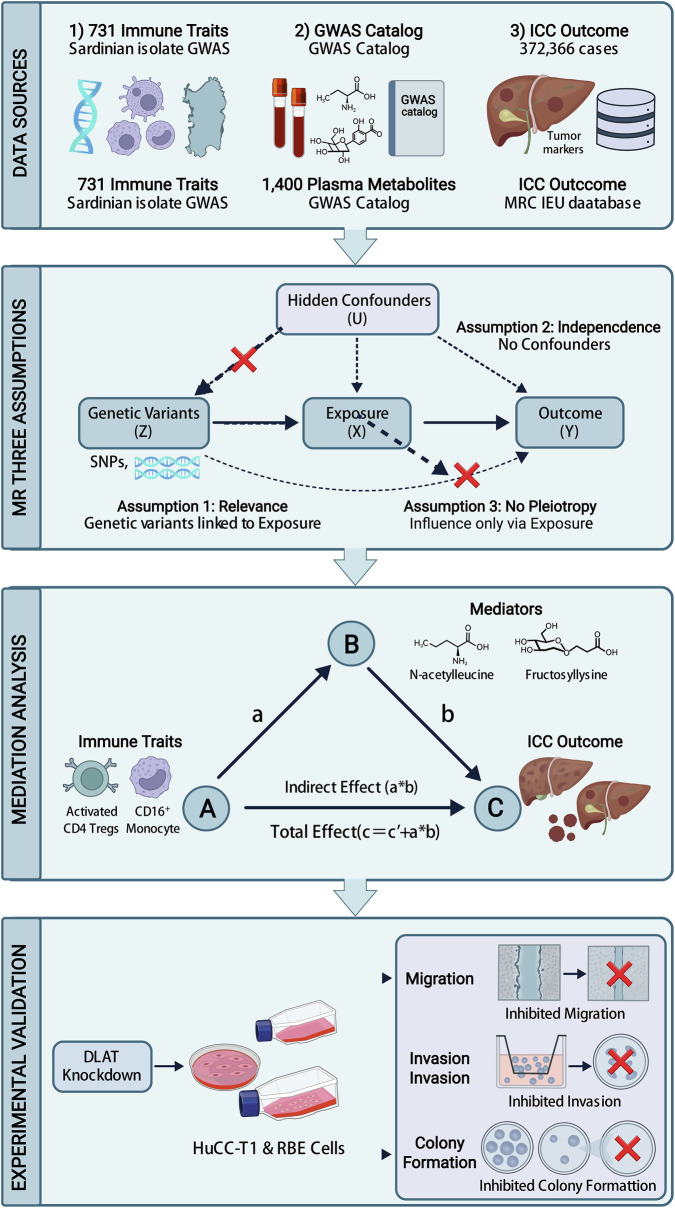
Mendelian Randomization Flowchart for the study. The overall impact of mediation analysis is split into two components: indirect and direct effects. Variable “a” represents the total influence of immune cell types on 1400 blood metabolites, while “b” signifies the impact of these metabolites on ICC. Variable “c” stands for the total effect of immune cell types on ICC. To calculate the indirect effect, use the formula: indirect effect = c - (a × b). The proportion of mediation is evaluated by dividing the indirect effect by the total effect.

We designed a study that included MR mediation analysis and experiments to explore the relationship between immune cells and ICC (Figure 1). First, we sourced genetic variants linked to immune traits from a large-scale GWAS study from a Sardinian isolate. We exclusively used non-overlapping datasets generated by multicolor flow cytometry. 731 distinct immune traits were categorized into four groups: absolute cell counts (AC, n = 118), median fluorescence intensities (MFI, n = 389), morphological parameters (MP, n = 32), and relative cell counts (RC, n = 192).

We sourced the ICC summary statistics from the MRC IEU OpenGWAS database. This massive GWAS meta-analysis included 372,366 cases and 372,016 controls. After strict quality control and data cleaning, we included about 7.7 million genetic variants in our MR analysis. We identified all ICC cases using the ICD-9 code 155.0. in this study (Kimberley Burrows, 2021).

We obtained summary data for 1,400 plasma metabolites from the GWAS Catalog (accession numbers GCST90199621 to GCST90201020). To maintain a consistent genetic background and reduce bias, we restricted our analysis to individuals of European ancestry (Chen et al., 2023).

There is no significant sample overlap across the samples.

### 2.2 Instrumental variables (IVs)

We ensured that each SNP met three core MR assumptions to reduce bias. First, each SNP must be strongly associated with the immune phenotype. Second, the instruments must be independent of any confounding factors. Finally, the SNPs must influence ICC risk only through the exposure pathway, with no evidence of horizontal pleiotropy (Richmond and Davey Smith, 2022; Smith and Ebrahim, 2004).

We selected SNPs with a significance threshold of 1 × 10^−5^ to ensure we captured all relevant variants. To eliminate linkage disequilibrium, we performed rigorous clumping with a window size of 10,000 kb and an r^2^ threshold of 0.001. For any SNPs missing from the outcome data, we utilized proxies with high linkage (r^2^ > 0.8) to ensure the genetic signal remained consistent. Finally, we excluded palindromic SNPs and filtered for an effect allele frequency between 0.3 and 0.7 to maintain data quality (Hemani et al., 2018). To evaluate the strength of our instruments and minimize weak instrument bias, we calculated the F-statistic (F = β^2^/se^2^) for each instrumental variable (Burgess and Thompson, 2011).

### 2.3 Statistics analysis

We performed all MR analyses in R using the “TwoSampleMR” package to ensure transparency and reproducibility. We used IVW, MR-Egger, and weighted median methods to analyze the causal links between immune profiles and ICC. We chose IVW as our primary method. A two-sided P < 0.01 was used to define statistical significance (Burgess et al., 2017). The MR-Egger method provides reliable results by adjusting for horizontal pleiotropy (Verbanck et al., 2018). Finally, the weighted median method improves accuracy by calculating the median effect across genetic variants (Hemani et al., 2018).

In order to maintain the validity of causal conclusions, multiple sensitivity studies were conducted to detect possible biases. We used Cochran’s Q test to check for heterogeneity among SNPs, with P < 0.05 as the threshold (Greco et al., 2015). To detect horizontal pleiotropy, we tested if the MR-Egger intercept was different from zero (Bowden et al., 2015). Finally, we used the MR-PRESSO global test to find and remove outliers (Verbanck et al., 2018).

### 2.4 Mediation analysis

We performed a two-step MR analysis to see if blood metabolites mediate the causal link between immune traits and ICC (Carter et al., 2021). We split the total effect of immune cells on ICC into direct and indirect parts to calculate the mediation ratio (Figure 1). The mediation was measured by finding the ratio of the indirect effect to the total effect. The Delta approach was used to get 95% confidence intervals (CIs) (Relton and Davey Smith, 2012).

### 2.5 Cell culture

We obtained HuCC-T1 and RBE ICC cell lines from Procell Life Science & Technology (Wuhan, China). Cells were cultured in RPMI-1640 medium supplemented with 10% FBS and 1% penicillin-streptomycin. The cultures were maintained at 37 °C with 5% CO_2_. The culture medium was replaced every two to three days.

### 2.6 RNA interference and transfection

HuCC-T1 and RBE cells were transfected with two specific siRNAs targeting DLAT: siRNA#1 (5′-CAG​CAA​CAT​TCG​TCG​GGT​TAT-3′) and siRNA#2 (5′-ATC​AAT​GAA​GGT​GAC​CTA​ATT​GC-3′), synthesized by Sangon Biotech. Transfection was performed using Lipofectamine 3000 (Invitrogen, L3000008) following the manufacturer’s protocol. RT-qPCR was used to check how well the knockdown worked 48 h after transfection.

### 2.7 Migration, invasion, and colony formation assays

To test cell motility, we seeded 4 × 10^4^ serum-starved cells in the upper chamber of Transwell inserts. We added medium with 20% FBS to the lower chamber as a chemoattractant. After incubation, the cells that migrated to the underside of the membrane were fixed and stained with crystal violet. In the subsequent phase, quantification was conducted by manually counting across numerous sectors using an inverted microscope.

An additional evaluation of cell motility was conducted in 6-well plates using a contact assay. In order to mitigate the confounding effect of cell division, the cells were pre-treated with 8 h of serum-free medium after they had reached a confluent monolayer. A uniform linear incision was generated using a 10 μL pipette tip. We photographed the incision at 0 and 24 h to monitor healing, after which used ImageJ to calculate the closure ratios.

To assess cell growth, we seeded ICC cells in 6-well plates (1,000 cells per well) and cultured them for 14 days. After 14 days, we washed the cells with PBS and fixed them in 4% paraformaldehyde for 30 min. Finally, we stained the cells with crystal violet and counted the colonies using ImageJ.

### 2.8 Ethics statement

This study analyzed publicly available GWAS summary statistics. All original studies obtained informed consent and IRB approval. As only de-identified, aggregated data were utilized, further ethical clearance was not required.

## 3 Results

### 3.1 The causal relationship between ICC development and immune phenotypes

**FIGURE 2:**
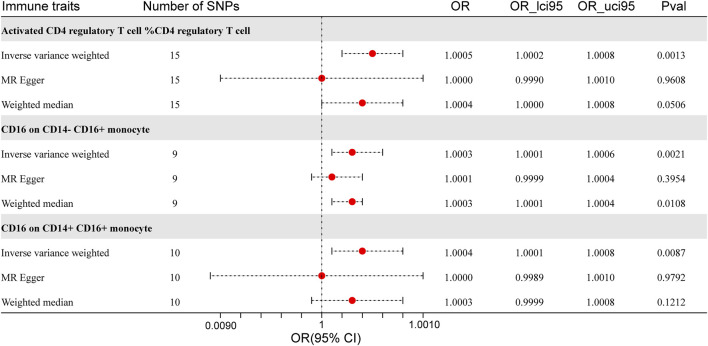
Forest Plot: Associations of immune cell with ICC risk.

Using the IVW method, we found three types of immune cells that were positively linked to ICC (Figure 2). Detailed information is presented in Supplementary Tables 3, 4. We used MR-Egger and MR-PRESSO tests to ensure our results were not affected by bias or outliers, and found no evidence of such issues (Supplementary Tables 5, 6).

An elevated proportion of activated CD4 regulatory T cells correlated with a heightened risk of ICC in the IVW technique (OR = 1.0005, 95% CI = 1.0002–1.0008, p = 0.0013), which was consistent in the weighted-median method (OR = 1.0004, 95% CI = 1.0000–1.0008, p = 0.0506). The MR-Egger intercept indicated no evidence of horizontal pleiotropy in the results (intercept p-value = 0.3117). The MR-PRESSO global test did not detect any putative pleiotropic SNP (p = 0.3300). We also analyzed other types of regulatory T cells, such as resting or total regulatory T cells. However, these subtypes did not show a significant causal link to ICC risk (all p > 0.05, Supplementary Table 3).

The expression of CD16 on the surface of CD14^−^CD16^+^ monocytes was favorably correlated with the probability of ICC in the IVW technique (OR = 1.0003, 95% CI = 1.0001–1.0006, p = 0.0021). The correlation between CD16 expression on CD14^−^CD16^+^ monocyte surfaces and the probability of ICC was maintained in both the weighted-median method (OR = 1.0003, 95% CI = 1.0001–1.0004, p = 0.0108) and the MR-Egger approach (OR = 1.0001, 95% CI = 0.9999–1.0004, p = 0.3954). The MR-Egger regression intercept indicated no evidence of horizontal pleiotropy (intercept p-value = 0.0698). The MR-PRESSO global test revealed no evidence of horizontal pleiotropy. p = 0.4300.

Additionally, ICC patients demonstrated elevated CD16 expression on the surface of CD14^+^CD16^+^ monocytes in the IVW method (OR = 1.0004, 95% CI = 1.0001–1.0008, p = 0.0087), which remained consistent in the weighted median method (OR = 1.0003, 95% CI = 0.9999–1.0008, p = 0.1212) and the MR-Egger method (OR = 1.0000, 95% CI = 0.9989–1.0010, p = 0.9792). No evidence of horizontal pleiotropy was seen in the MR-Egger regression (intercept p-value = 0.3923) and the MR-PRESSO global test (p = 0.4930).

Our findings remained consistent across different analysis methods, which makes the causal links we identified more reliable (Supplementary Tables 3, 4). We found very little variation among the genetic tools used for immune cells, indicating that our results are stable (Supplementary Table 7). It emphasizes the disease’s complexity and the importance of fully understanding the immune system’s function.

### 3.2 Proportion of the association between ICC and immune traits mediated by metabolites

**FIGURE 3:**
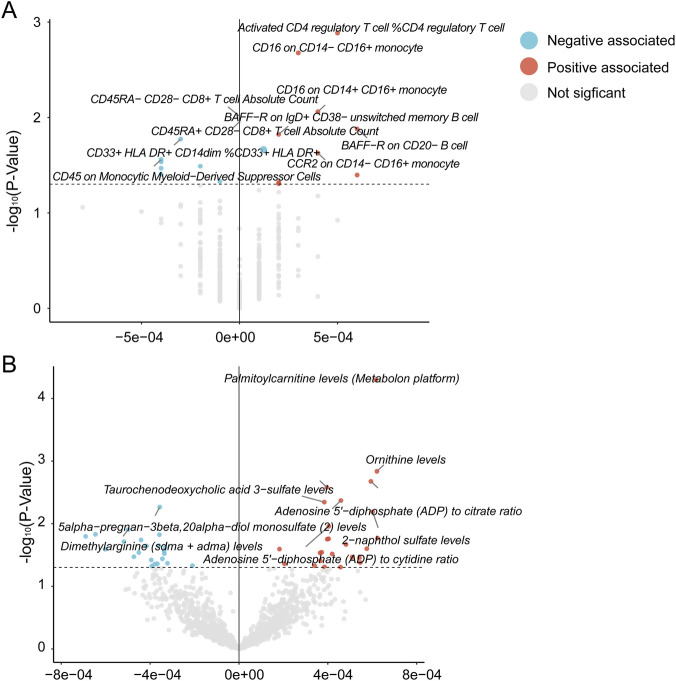
Volcano plots of causal associations with ICC risk. These plots illustrate the causal effects of various immune traits **(A)** and circulating blood metabolites **(B)** on the development of intrahepatic cholangiocarcinoma (ICC).

**FIGURE 4:**
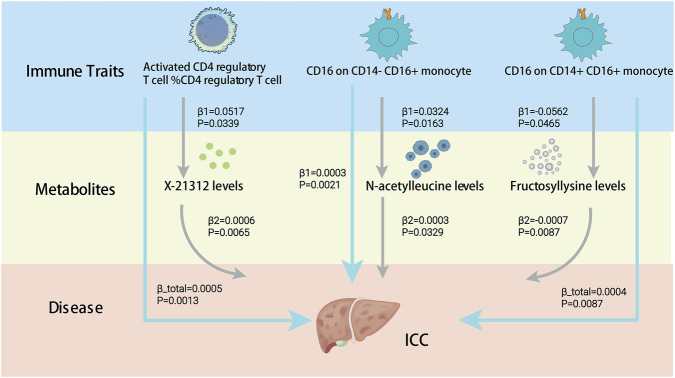
Causal effects of immune cells on ICC mediated by metabolites. β refer to the causal effect estimates using the IVW.

The activation of CD4 regulatory T cells, CD16 on CD14^−^ CD16^+^ monocytes, and CD16 on CD14^+^ CD16^+^ monocytes favorably influences the incidence of ICC. This indicates that the expression of CD4 in T cells and CD16 on monocytes may be critical molecules implicated in this process. To understand this better, we performed a mediation analysis to see how immune traits and blood metabolites relate to ICC (Figure 3). Our results identified specific metabolites that act as a bridge between immune cells and ICC risk (Figure 4).

We identified N-acetylleucine levels as a putative mediating molecule for the expression of CD16 on CD14^−^ CD16^+^ monocytes. N-acetylleucine concentrations were identified as a risk factor for ICC, contributing 2.8441% to the heightened risk of ICC. Likewise, for the expression of CD16 on CD14^+^ CD16^+^ monocytes, we identified Fructosyllysine levels as a potential mediating factor. Fructosyllysine levels were identified as a risk factor for ICC, contributing 8.4252% to the heightened risk of ICC.

### 3.3 Knockdown of DLAT inhibited cell proliferation and metastasis of ICC

**FIGURE 5:**
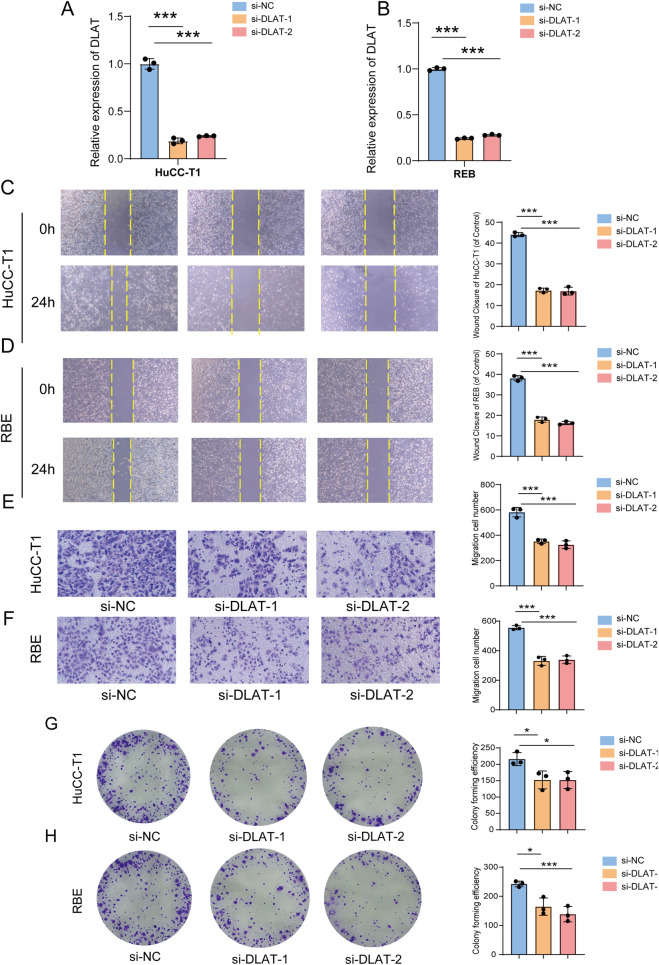
Impact of DLAT Knockdown on ICC Progression. **(A,B)** RT-PCR was used to verify DLAT knockdown in HuCC-T1 and RBE cells. **(C,D)** Wound healing assay. **(E,F)** Transwell migration assay. **(G,H)** Colony formation assay. *p < 0.05 and ***p < 0.001.

MR mediation analysis identified N-acetyl-leucine—a synthetic derivative infrequently documented to occur in vivo—as a crucial plasma metabolite connecting immune-cell characteristics to IHC risk. N-Acetylleucine is a prodrug of leucine (Churchill et al., 2021). It is converted into L-leucine through intracellular deacetylation. Directly manipulating this derivative *in vitro* is methodologically challenging. Therefore, we targeted DLAT to investigate how the broader leucine metabolic axis influences the development of ICC (Vesnina et al., 2026; Wang et al., 2025). In this study, we utilized RNA interference to downregulate the gene. RT-qPCR validated effective silencing in HuCC-T1 and RBE cells (Figures 5A,B).

The wound-healing assay demonstrated that the silencing of DLAT nearly entirely inhibited the mobility of HuCC-T1 and RBE cells (Figures 5C,D). The identical knockdown concurrently diminished their invasive capability (Figures 5E,F). Cells with DLAT knockdown exhibited a significant decrease in colony formation compared to the negative controls (Figures 5G,H). Collectively, our results suggest that inhibiting DLAT suppresses the growth and migration of ICC cells.

## 4 Discussions

ICC is a highly lethal malignancy (Jiang et al., 2024). While immune cells are known to drive tumor progression, their specific roles in ICC remain largely unexplored (Li et al., 2025; Feng et al., 2025; Lei et al., 2020). Using MR analysis, we identified a clear causal link between three immune traits and ICC susceptibility. We found that activated CD4 regulatory T cells are a consistent risk factor. Similarly, CD16 surface expression on both CD14^−^CD16^+^ and CD14^+^CD16^+^ monocytes increases ICC risk. These findings suggest that these immune subsets may drive disease progression. Furthermore, we identified N-acetylleucine and fructosyllysine as key metabolic mediators. Our work provides new insights into the mechanisms of occurrence of ICC.

Studies showed that the anti-tumor response and patient survival were closely linked to the activation of tumor-specific CD4 regulatory T cells (Jackaman et al., 2012). Previous experiments demonstrated that the co-cultivation of CD4^+^ T cells differentiated into regulatory T cells, extracted from peripheral blood, with cholangiocarcinoma cells resulted in the aggregation of regulatory T cells, leading to an increase in the number of malignant cholangiocarcinoma cells (Zhang et al., 2023). These findings are consistent with our research results, as we have also observed an increased risk of ICC development with an increasing percentage of activated CD4 regulatory T cells. It is interesting that only the “activated” subset of CD4 regulatory T cells increases ICC risk. This may be because activated Tregs have much stronger power to suppress the body’s immune response against tumors compared to resting ones.

Furthermore, it is worth noting that our study further revealed that increased expression of CD16 on CD14^−^CD16^+^ and CD14^+^CD16^+^ subsets of monocytes is positively associated with the occurrence of ICC. Previous research showed that CD16 was a type of Fcγ receptor (FcγR) that bound to IgG molecules through its Fc portion. Experimental evidence also suggested that the expression of CD16 was significantly expanded in various pathological conditions (Ancuta et al., 2003). CD16^+^ monocytes exhibited pro-inflammatory and tumor-promoting characteristics. They generated significant quantities of TNF-α, IL-1β, and VEGF. When recruited into tumour tissues, they developed into tumor-associated macrophages (TAMs). Patients with cholangiocarcinoma had higher circulating numbers of CD14^+^CD16^+^ monocytes. These elevated levels were associated with tissue invasive characteristics and greater TAM infiltration. These data provided evidence that CD16^+^ monocytes were important drivers of ICC development (Subimerb et al., 2010; Genc et al., 2003).

In this study, we made an exciting discovery that the increased expression of CD16 on CD14^−^CD16^+^ and CD14^+^CD16^+^ monocyte subsets was positively associated with the occurrence of ICC, with N-acetylleucine levels acting as a mediating molecule in this process. Our MR mediation analysis suggests that immune cells may significantly increase the risk of ICC by increasing N-acetylleucine levels. N-acetylleucine is an N-acetylated derivative of leucine synthesized mainly through acetylation reactions. The potential application of N-acetylleucine in neurodegenerative diseases is gradually gaining attention (Oertel et al., 2024; Tifft, 2024).

Direct manipulation of N-acetylleucine in the lab is difficult because it is an inactive form that turns into L-leucine inside liver cells. We focused on earlier steps in this process and targeted DLAT, a key controller of leucine balance, to see if changing this pathway matched the risk patterns from our analysis. DLAT is a part of a larger enzyme machine. It helps make a fuel molecule from pyruvate to power the cell’s energy cycle. The breakdown of leucine also produces this same fuel molecule for the energy cycle. In addition, breaking down leucine needs a common helper molecule to accept nitrogen. This showed an important link between sugar and protein metabolism, which was controlled by the DLAT-leucine connection (Vesnina et al., 2026; Wang et al., 2025). Recent studies showed that DLAT was an epigenetic regulator that helps many cancers grow by blocking the breakdown of leucine. This process silenced genes that normally stop tumors, a mechanism seen in both ICC and other cancer types. DLAT also links metabolism to cuproptosis, a form of cell death. Consistent with our hypothesis, we discovered that DLAT knockdown reduced the proliferation, migration, and invasion of ICC cells by depleting intracellular leucine levels. The findings of the N-acetylleucine and DLAT axis suggest a ‘druggable’ vulnerability in ICC. Similar to our metabolic findings, certain piRNAs were shown to regulate metabolism and enhance treatment efficacy by modulating signaling pathways such as the PI3K/AKT/mTOR axis (Deng et al., 2024). Pharmacological targeting of this metabolic system is a possible treatment option. Clinical strategies, such as metabolic or dietary leucine restriction, may be investigated to increase the efficacy of current treatments.

A recent study identified an increase in the effector CD8^+^CD45RA+CD28^−^ T cell fraction in cervical cancer patients (Pilch et al., 2002). Elevated CD28^−^CD8^+^ T cells were linked to chronic immune activation (Strioga et al., 2011). These T cell subtypes were common in human cancers, where they often suppressed T cell growth and impaired cytotoxic functions (Filaci et al., 2007; Tsukishiro et al., 2003). The findings of this investigation align with those of the present study. Our mediation analysis suggests that higher fructosyllysine levels may lower ICC risk. Conversely, CD16^+^ monocytes appear to increase ICC risk by lowering these fructosyllysine levels.

Although the genetic risk we identified is small, the large sample size in this study provides good evidence for a causal association. In MR research, these findings represent the long-term, lifelong impact of genetic factors rather than sudden medical changes. However, our study has several limitations. First, the immune traits were obtained from a Sardinian cohort. This is a genetically isolated population with distinct environmental and physiological conditions. This may limit how well our findings apply to other ethnic groups. Second, we did not perform direct metabolite measurements or rescue experiments using leucine or N-acetylleucine. Such experiments would strengthen our findings. Finally, further studies using metabolite verification are needed to identify how leucine derivatives change inside cells.

## 5 Conclusion

This study used two-sample MR to link specific immune traits to ICC risk. Our mediation analysis identified N-acetylleucine and fructosyllysine as key metabolic bridges. These findings underscore the therapeutic potential of metabolic intervention in ICC. While further research is needed to clarify the underlying biological mechanisms, our work provides a foundation for developing early interventions and targeted treatments for ICC.

## Data Availability

The datasets presented in this study can be found in online repositories. The names of the repository/repositories and accession number(s) can be found below: https://www.jianguoyun.com/p/DaShqhEQ5KiPDhi0gJwGIAA.
